# Ability of Soil Isolated Actinobacterial Strains to Prevent, Bind and Biodegrade Ochratoxin A

**DOI:** 10.3390/toxins9070222

**Published:** 2017-07-14

**Authors:** Rachelle El Khoury, Florence Mathieu, Ali Atoui, Hiba Kawtharani, Anthony El Khoury, Charbel Afif, Richard G. Maroun, André El Khoury

**Affiliations:** 1 Laboratoire de Mycologie et Sécurité des Aliments (LMSA), Centre d’analyse et de Recherche (CAR), Campus des Sciences et Technologie, Université Saint-Joseph, Dekwaneh-Beyrouth 1104-2020, Lebanon; rachelle.khouryel@net.usj.edu.lb (R.E.K); hiba.kawtharani@net.usj.edu.lb (H.K.); andre.khoury@usj.edu.lb (A.E.K.); charbel.afif@usj.edu.lb (C.A.); richard.maroun@usj.edu.lb (R.G.M); anthony.khoury4@net.usj.edu.lb (A.E.K.); 2 Laboratoire de Génie Chimique, CNRS, INPT, UPS, Université de Toulouse, Toulouse 31 326, France; florence.mathieu@ensat.fr; 3 Laboratory of Microbiology, Department of Natural Sciences and Earth, Faculty of Sciences I, Lebanese University, Hadath Campus, P.O Box 5 Beirut, Lebanon

**Keywords:** Ochratoxin A, actinobacteria, biocontrol, binding, detoxification, genes expression

## Abstract

Ochratoxin A (OTA) is one of the most important mycotoxins, and contaminates several agricultural products, particularly cereals, grapes, maize, barley, spices and coffee. The aim of this project was to reduce the levels of OTA by supplementing the artificially contaminated solutions with seven strains of actinobacteria (AT10, AT8, SN7, MS1, ML5, G10 and PT1) in order to evaluate their capacity for binding and metabolizing the OTA, as well as their ability to reduce the expression of the genes responsible for its production in *A. carbonarius*. In the first part of this study, we evaluated the capacity of *Streptomyces* strains for binding OTA on their surfaces after 0, 30 and 60 min of incubation with PBS solution supplemented with OTA. In the second part, we tested the ability of these strains, as well as their supernatants, to detoxify the ISP2 medium. Finally, we studied the effect of the *Streptomyces* cocultured with *Aspergillus carbonarius* on the expression of OTA biosynthesis genes. Results showed that, among the strains co-cultured with *A. carbonarius*, the strain G10 was able to reduce the expression of *acpks*, *acOTApks*, *acOTAnrps* and *vea* genes, thus reducing OTA from solid PDA medium to 13.50% of reduction. This strain was remarkably able to detoxify and bind OTA up to 47.07%. Strain AT8 was stronger in detoxifying OTA (52.61%), but had no significant effect on the studied gene expression.

## 1. Introduction

Ochratoxin A (OTA) is a secondary metabolite produced by molds of the genera *Aspergillus* and *Penicillium* on several food commodities in pre- and post-harvest conditions [1,2,3]. The dangerous effects of OTA on human health (carcinogen group 2B) [4], as well as its impact on economic losses [5], made the prevention of production of this toxin and/or its detoxification from food an important task for the scientific community. Several biocontrol approaches have been elaborated to prevent the fungal growth or production of OTA with plant extracts or, more particularly, essential oils and phenolic compounds [6,7,8]. Others have been focused on treating the contaminated food with living organisms such as lactic acid bacteria (LAB) and yeasts, to reduce the OTA concentration. This biological decontamination is either performed by OTA biodegradation using microorganisms and their enzymes [9,10,11], or by binding the toxin on the cell wall surface of these microorganisms [12,13,14]. Both approaches help reduce OTA without significant losses in nutritive values, since no harmful chemicals are used in this treatment of contaminated food [15,16,17,18]. For instance, *Lactobacillus bulgaricus* is able to biodegrade 94% of the OTA added to the cultures after 24 h of incubation [19]. Similarly, *Saccharomyces cerevisiae* eliminated 41% of OTA via enzymatic degradation after 24 h at 30 °C. On the other hand, Turbic, et al. [20] claimed that OTA reduction by *Lb. rhamnosus* strains (76% of reduction) was caused by the adsorption of this toxin onto the LAB cell walls, since the acid-treated bacteria were more effective for removing OTA than the viable ones after 2 h at 37 °C.

*Actinobacteria* are Gram-positive bacteria that can be found in soil, and contain *Streptomyces*; one of the largest of bacterial genera. *Streptomyces* and other actinobacteria are major contributors to biological buffering of soils, and are a source of many antibiotics [21,22]. The antifungal and antimycotoxigenic effects of actinobacteria were tested by Verheecke, et al. [23], when they evaluated the effect of 16 strains of *Streptomyces* on aflatoxin B (AFB) detoxification, and found that reduction levels ranged between 15.6% and 82.2% for the tested strains. When they further investigated the reason behind these reductions, they found that AFB inhibitions were not related to adsorption, but rather to the repression of AFB biosynthesis genes in *A. flavus.* Nevertheless, the ability of *Streptomyces* to reduce OTA has not been yet established. Hence, the aim of this study is to evaluate the effect of seven *Streptomyces* strains, named AT10, AT8, SN7, MS1, ML5, G10 and PT1, on OTA levels, and investigate the mode of action of each strain—i.e., whether they degrade OTA or bind it to their cell walls—as well as their effect on the expression of the genes responsible for OTA biosynthesis (*acpks*, *acOTApks*, *acOTAnrps*) and the regulatory genes (*veA* and *laeA*) in *A. carbonarius* S402.

## 2. Results

### 2.1. Kinetics of OTA Binding to Actinobacterial Cell Wall

The PBS contaminated with 45.12 ng/mL of OTA and inoculated with the tested bacterial strains was examined after 0, 30 and 60 min for the remaining OTA concentration. Reductions were all significant compared to the control. Nevertheless, the levels were not the same for all the strains (Table 1). For example, AT10, AT8, SN7, G10 and PT1 were the most effective strains, as they produced reductions of 25.62, 16.07, 33.93, 16.23 and 24.85%, respectively, after 60 min of incubation. Levels of inhibition were higher at 60 min than at 30 min for all the strains. Since this study was handled in a non-nutritive solution (PBS), the observed reductions could not be attributed to an enzymatic activity of the actinobacteria. Moreover, the sums of the reduced OTA with the washed OTA extracted from the pellets were equal to the initial concentration (45.12 ± 1.2). This observation confirms that the reduced OTA is only due to the ability of the strains to bind OTA onto their cell walls.

### 2.2. Detoxification of OTA by Actinobacterial Strains and Their Supernatant

After the culture of each actinobacterial strain in ISP2 liquid medium at 28 °C for 5 days, their supernatant was added to ISP2 medium containing 95.45 ± 1.7 ng/mL of OTA (initial concentration). After the incubation period (5 days at 28 °C), results (Table 2) showed that all the supernatants have to ability to degrade the OTA initially added to the cultures. More particularly, the highest degradation levels were obtained with AT8 supernatant (22.42%) after 5 days of culture. On the other hand, PT1 supernatant was only able to decrease 14.08% of the initial OTA concentration, while the supernatants of SN7, MS1 and G10 strains reduced it to a similar degree (16.08%, 16.83% and 16.46% respectively for each strain). Nevertheless, the culture of the actinobacterial strains with 95.45 ± 1.7 ng/mL of OTA in ISP2 liquid medium (5 days at 28 °C) resulted in higher reductions. The lowest OTA concentrations were found when AT10 and SN7 were added to the medium, with 51.94% and 52.68% reductions, respectively, more than 2.4 and 3.2 times that achieved by their corresponding supernatants. The lowest detoxification activity was obtained with the MS1 strain, reducing OTA by 22.83% only. Statistically, AT8 and PT1 had similar reduction activity (42.13% and 44.97%).

### 2.3. Effect of Actinobacteria on A. carbonarius Dry Weight, OTA Production and Genes Expression

After the coculture period of *A. carbonarius* S402 and the studied strains of *Streptomyces* the effect of each strain on the dry weight of *A. carbonarius*, its radial growth and OTA production are represented in Table 3.

Results showed that each strain of *Streptomyces* affected *A. carbonarius* S402, whether the effect as on its dry weight, radial growth and/or OTA production. For instance, PT1 reduced the radial growth of *A. carbonarius* (52.2%), and resulted in a reduction of dry weight (32.02%) and OTA production (11.69% of reduction). On the other hand, MS1 reduced OTA by 23.91% (the highest reduction in this study) and reduced the dry weight by only 7.1%, without significantly affecting the radial growth. The strains ML5 and G10 reduced OTA to a similar degree (13.70% and 13.50%, respectively), while AT10, AT8 and SN7 had no significant effect on the OTA production. In order to better understand these differences in OTA reduction, we further investigated the expression of the genes involved in OTA biosynthesis (*acpks*, *acOTApks*, *acOTAnrps*, *veA* and *laeA*) in *A. carbonarius* S402 (Table 4).

The OTA biosynthesis genes (*acpks*, *acOTApks*, *acOTAnrps*), as well as the regulatory genes (*laeA* and *veA*), in *A. carbonarius* were affected by the presence of the different tested strains. The strains MS1, G10 and ML5 had a greater impact on the repression of the three biosynthesis genes *acpks* (37.1, 9.0 and 39.0%, respectively, for each strain), *acOTApks* (23.9%, 18.3% and 23.0%, respectively) and *acOTAnrps* (21% for MS1, and 11.9% for both G10 and ML5). These gene repressions were in correlation with the OTA reductions described in Table 4. Other strains, such as AT10, AT8, SN7 and PT1 had no significant effect on the expression of those genes. As for the regulatory genes, the expression of *laeA* was not regulated by any of the strains. On the other hand, it was slightly downregulated by SN7 and PT1 (3% for both strains, equally), and MS1 (increased by 9%), while the 4 other strains (AT10, AT8, G10 and ML5) had no significant effect on *laeA*. The expression of the second regulatory gene *veA* was downregulated by AT8, SN7, MS1, PT1 and G10 by 2.0, 10.0, 11.4, 10.0 and 7%, respectively, for each strain. Neither AT10 and ML5 affected the expression of this gene. These results showed that the downregulation in the expression of the biosynthesis genes, caused by MS1, ML5 and G10 strains, induced a reduction in the OTA production. As for the strains that did not significantly affect the expression of the studied genes, their presence in the culture medium did not alter the production of OTA by *A. carbonarius.*

## 3. Discussion

Preventive OTA techniques should, in some cases, be performed with detoxification approaches, in order to fully reduce the levels of this toxin from food and feed. This study was intended for this purpose, by testing different strains of actinobacteria in terms of their ability to bind, degrade and prevent OTA production in in vitro conditions. Lactic bacteria and yeasts are known for their ability to bind diverse molecules, such as killer toxins and metal ions, to their cell wall surfaces [12,13,14]. In the first part of this study, we investigated the capacity of seven actinobacterial strains to bind OTA to their surfaces. Results showed that each of the seven strains was capable of binding OTA, but with different levels. Indeed, the strain SN7 recorded the highest binding capacity (33.93%) after 60 min of contact. AT10 and PT1 were equally effective in reducing OTA, with reductions of 25.62% and 24.85%, respectively. Similarly, AT8 and G10 decreased OTA concentration from 45.12 ng/mL to 37.92 and 37.82 ng/mL, respectively. The others, MS1 and ML5, had only a slight impact on binding OTA to their walls. This type of detoxification was observed with heat- and acid-treated *Lacobacillus rhamnosus* strains and oenological *Saccharomyces* strains that were more effective at removing OTA than viable cells in PBS [11,20]. Piotrowska and Zakowska [24] found that 1 mg of OTA was decreased by 70% and 87% with *L. acidophulis* and *L. rhamnosus*, respectively, after 5 days at 37 °C. The binding mechanism of OTA and other mycotoxins by bacterial strains has been explained by several authors. Pereyra, et al. [25] tested two yeast wall cells (YCW1 and YCW2) for their ability to bind OTA, both containing mannans and β-glucans, but with different percentages (21% and 5.9% of mannans and 23% and 17.4% of β-glucans for YCW1 and YCW2, respectively). These authors noticed that these differences in polysaccharide did not affect the ability of those components to bind OTA. Similar results were obtained by Yiannikouris, et al. [26], who studied the ability of four cell walls, which had different compositions on binding OTA, and reported that there were no changes in the amount of OTA adsorbed. The mechanism of OTA elimination by binding with microorganisms is a rapid process. Indeed, it was found that a period of half an hour of contact between LAB cells and OTA was enough to remove more than 80% of the total OTA removed throughout the experiment, which lasted 24 h, and no major changes were observed by the end of the experiment [27]. These observations were similar to those obtained by El-Nezami, et al. [28], who found that even at 0 min, 80% of the AFB1 had been removed by two strains of *L. rhamnosus*. The binding mechanism of OTA with actinobacterial strains has not been yet elucidated. Nevertheless, it was thoroughly explained for Gram-positive bacteria, a group of which actinobacteria are a part. In fact, Piotrowska [27] found that cells that had had their cell wall partially removed bound the OTA less than non-modified cells, which proves the importance of the cell walls in this type of detoxification. Moreover, *Escherichia coli*, Gram-negative bacteria, was found to be unable to bind toxins, which was attributed to its moderately hydrophilic surface (rich in lipopolysaccharides). While LAB bacteria have a similar wall composition, they have hydrophobic pockets on their wall surfaces that confer the bacteria with the ability to bind OTA [29].

In this study we found that the supernatant of each of the tested strains had a slight but significant effect on OTA reduction in ISP2 liquid medium after 5 days of incubation. Reduction levels were even higher when the strains were cultured in ISP2 liquid medium contaminated with 95.45 ng/mL of OTA. Indeed, AT8 supernatant was capable of degrading 22.42% of the OTA, while the strain itself reduced 42.13% of the added OTA. Some authors have assumed that biodegradation of OTA is also involved in the food detoxification process. Viable *L. acidophilus* cells were able to reduce OTA (95% after 4 h) more efficiently than non-viable cells [30]. Similarly, *S. cerevisiae* was able to degrade 38% of this toxin after 24 h, without any descriptions on the type of the resulting metabolites [10]. Schatzmayr, et al. [31] explained this process when they noticed that *Trichosporum*, *Rhodotorula* and *Cryptococcus* strains biodegraded OTA through the hydrolysis of the amide bond, resulting in OTα and L-*β*-phenylalanine accumulation. This type of degradation mechanism can be adopted in detoxification techniques, since both of the resulting metabolites are virtually non-toxic. Other studies suggested that biodegradation can be achieved via the cleavage of the lactone ring, resulting in the formation of an opened lactone form of OTA that exhibits similar toxicity of OTA in rats [9].

In the third part of this study, results showed that MS1, G10 and ML5 strains had a significant impact on the expression of *acpks*, *acOTApks* and *acOTAnrps* in *A. carbonarius* S402, that eventually resulted in the reduction of OTA production, reducing production by 23.91%, 13.50% and 11.69%, respectively, for each strain, whereas the coculture of *A. carbonarius* S402 with the other 4 strains (AT10, AT8, PT1 and SN7) did not affect the expression of these genes. This type of mode of action was studied by Verheecke, et al. [32] on *A. flavus* and *A. parasiticus*, and it was found that some actinobacterial strains (S27 and S17) did not significantly affect the expression of AFB biosynthetic genes, while others (S06, S13 and S35) had a great impact on several genes (S06 downregulated *aflS* expression by 100%).

This study shows that the application of different actinobacterial strains might reduce the OTA contamination, since each strain acted differently when it was put into contact with the toxin. OTA detoxification (binding and biodegradation) with actinobacterial strains might be more effective than preventing the production of this toxin. Further studies are required on this topic, in order to evaluate the effect of the studied bacteria on the integrity of the decontaminated food.

## 4. Materials and Methods

### 4.1. Actinobacteria and A. carbonarius Strains

Several actinobacterial strains, previously isolated from Algerian soils, were chosen for our study. In order to revive these strains (AT10, AT8, SN7, MS1, ML5, G10 and PT1), they were first sub-cultured for 4 days at 28 °C on an ISP2 (International *Streptomyces* Project 2) agar medium (4.0 g yeast extract, 10.0 g malt extract, 4.0 g dextrose, 20.0 g agar, 1 L tap water, pH adjusted to 7.2). In parallel, an *A. carbonarius* spore solution was prepared in the same manner described in our previous work [8]. The spore count was evaluated with a Neubauer haemocytometer (Superior, Marienfield, Lauda-Konigshofen, Germany); the concentration was adjusted to 10^6^ spores/mL and kept at 4 °C before use.

### 4.2. Preparation of Ochratoxin A contaminated PBS

The OTA standard (Supelco, Bellefonte, PA, USA) was suspended in acetonitrile (Sigma-Aldrich, 3050 Spruce Street, St. Louis, MO, USA) in order to prepare a stock solution of 3.0 μg/mL. Afterwards, a working solution of 95.45 ng/mL was prepared by diluting the stock solution with PBS. In order to evaporate the non-aqueous phase, an evaporation step at 45 °C with nitrogen is essential at this point in order to dissolve the OTA droplets in PBS. This solution was later used for binding and detoxification essays.

### 4.3. Binding Assay 

#### 4.3.1. Preparation of Actinobacteria Suspension

Each strain of actinobacteria was cultured in 20 mL of liquid ISP2 medium for 5 days at 28 °C in a shaker/incubator (Model SI-45, serial number AS-SI45-1038E, Trading Company, Ningbo, Zhejiang, China). The bacterial solution was prepared by filtering the cultures through Sartorius 150 mm filters (grade 290, particles retention size 80 g/m^2^, Sartorius filter discs, AG-37070 Goettingen, Germany) (the filtrated ISP2 medium was kept for further use). The filters were then placed in sterile pots containing each 10 mL of sterile water. The pots were vigorously shaken to separate the actinobacteria from the filters. The bacterial solution was directly used for the binding tests.

#### 4.3.2. OTA Binding Kinetics

In 2 mL tubes, 1 mL of the OTA contaminated PBS was added to 1 mL of bacterial suspension (OTA final concentration of 45.12 ng/mL). At 0, 30 and 60 min, tubes were centrifuged and 1 mL of the supernatant was purified with Ochraprep Immunoaffinity columns and quantified with Waters Alliance HPLC system (Milford, MA 01757 USA)). As for the pellets, they were washed with methanol acidified with acetic acid (98:2 *v/v*), purified with Ochraprep Immunoaffinity columns (R-Biopharm, Glasgow, UK) and quantified with HPLC.

### 4.4. Detoxification Activity of the Actinobacteria

The spores of the *actinobacterial strains* were scrapped from the pre-culture surfaces and added to 20 mL of liquid ISP2 medium, artificially contaminated with OTA solution. The total concentration of OTA was adjusted in order to have 95.45 ng/mL of OTA for each condition. The cultures were held in a shaker/incubator (Model SI-45, serial number AS-SI45-1038E) at 28 °C for 5 days with a constant shaking speed of 250 rpm. After the incubation period, the culture tubes were shaken and then centrifuged at 13,000 rpm. A volume of 1 mL of the supernatant was removed and purified with Ochraprep Immunoaffinity columns (R-Biopharm, Glasgow, UK) following the manufacturer’s protocol, and analyzed by HPLC.

In order to evaluate the strains capacity on OTA detoxification, we proceeded to follow the previously described protocol. However, the OTA-contaminated ISP2 medium was inoculated with the supernatants of the studied actinobacteria instead of the bacterial solutions.

### 4.5. Coculture of A. carbonarius and Actinobacteria Study

**Figure 1:**
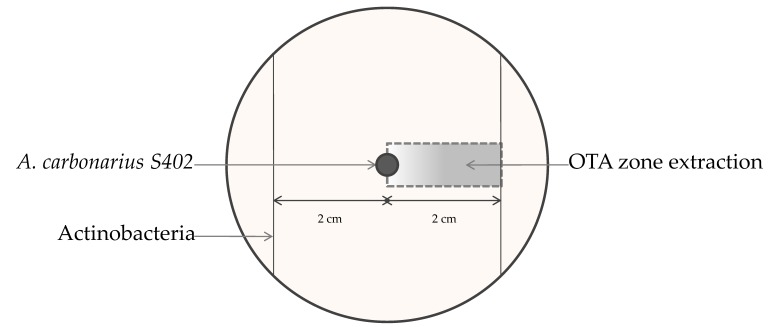
Schematic representation of actinobacteria and *A. carbonarius* S402 cocultures on Potato Dextrose Agar (PDA) for 5 days at 28 °C.

Each of the studied *actinobacterial strains* was co-cultured with *A. carbonarius* in Potato Dextrose Agar medium (PDA) (Biolife, Italiana S.R.L, viale Monza, Milano, Italy) by placing two parallel lines, each 2 cm away from the center of the Petri dishes (Figure 1). The center of the medium was inoculated with 10^6^ spores of *A. carbonarius* S402. The cultures were then held at 28 °C for 5 days.

#### 4.5.1. Effect on Growth of *A. carbonarius*

In order to evaluate the dry fungal weight, a sterilized transparent cellophane film was placed on the surface of the PDA medium prior to its inoculation with *A. carbonarius* and the actinobacterial strains. After the coculture period, the antifungal activities of each actinobacterial strain were evaluated by measuring the radial growth (cm) and the dry weight (g) of *A. carbonarius* S402, both after 5 days of culture. *A. carbonarius* S402 dry weight was evaluated by cutting the cellophane film around the fungus and weighing the mycelium after its desiccation (placing the transparent film carrying the culture at 100 °C for 24 h). As for the radial growth, diameter measurements of *A. carbonarius* cultures were taken after 5 days of incubation at 28 °C.

All assays were carried out in triplicate for each coculture condition.

#### 4.5.2. Total RNA Extraction, cDNA Synthesis and qRT-PCR for Gene Expression Analysis

Coculture conditions of *A. carbonarius* S402 and actinobacteria were the same as described previously. After the culture period, the mycelium was cut carefully from the rest of the cellophane film and ground into fine powder with liquid nitrogen. The total RNA was then extracted from the powder using RNeasy kit (Qiagen, Düsseldorf, Germany) following the manufacturer’s protocol. The RNA was then treated with DNase I Amplification Grade (Sigma-Aldrich, St. Louis, MO, USA). Before performing cDNA synthesis, the quantity and integrity of the extracted RNA were evaluated using the Experion RNA analysis kit and software (version 3.20, 2015, BioRad, Marnes-la-Coquette, France). The synthesis of the single strand cDNA was performed starting with 1 µg of total RNA using Advantage RT-for-PCR kit with both oligo (dT) and random hexamer following the manufacturer’s protocol (Clontech, Mountain View, CA, USA).

As for the qRT-PCR analysis, the expression of the targeted genes (*acpks*, *acOTApks*, *acOTAnrps*, *laeA* and *veA*) was evaluated with CFX96 Touch Real Time PCR detection system BioRad (version 3.0, 2012, Hercules, CA, USA), using SsoAdvanced Universal Sybr Green Supermix (Bio-Rad, Marnes-la-Coquette, France), starting with 100 ng/µL of the synthesized cDNA and following the manufacturer protocol for the mix preparation and amplification cycles. The Tm (melting temperature) was optimized and used at 58 °C. The reference gene, *β-tubulin*, was chosen based on our previous study [8]. The efficiencies of the primers (Table 5) were determined by a series of cDNA dilutions, and were calculated from the slopes of the obtained curves.

### 4.6. OTA Extraction

The OTA concentration was performed by extracting 3 agar plugs from the PDA medium, with 0.5 cm diameter each. OTA was extracted by adding 1 mL of methanol and shaken for 1 h. After the incubation period, the mix was centrifuged at 13,000 rpm (revolutions per minute) for 20 min. The supernatant containing the OTA was then purified with Ochraprep immunoaffinity columns (R-Biopharm, Glasgow, UK) following the manufacturer’s protocol.

### 4.7. HPLC Analysis

The concentration of the purified OTA was evaluated with the Water Alliance HPLC system using an Utisphere ODB column, C18 (150 × 4.6 mm, 5 µm, 120 Å) (Interchim, Montluçon, France) at 30 °C. The isocratic flow (flow rate of 1 mL/min) was composed of 49% acidified water (0.2% of acetic acid) and 51% acetonitrile for 30 min of run. The volume of the injected extract was set at 100 µL, and OTA was detected after 8 min of run by a fluorescent detector at 333/440 nm excitation/emission wavelengths.

### 4.8. Statistical Analysis

All the data in this study were analyzed through One-Way Analysis Of Variance (ANOVA) and paired t-test using STATGRAPHICS Centurion XVI (version 16.1.11 for Windows, 2010, StatPoint Technologies Inc., Warrenton, VA, USA).

## Figures and Tables

**Table 1:** Kinetics of OTA binding to actinobacterial strains in PBS solution for 0, 30 and 60 min.

Strains	Time (min)	OTA Concentration in PBS (ng/mL) (% of Reduction)	OTA Concentration after Bacterial Pellet Washing (ng/mL)
**Control**	0	45.12 ± 1.2 ^a^	
**AT10**	0	39.59 ± 1.1 *^,e^ (12.37)	5.53 ± 0.12
	30	38.19 ± 0.9 *^,d^ (15.46)	6.82 ± 0.09
	60	33.60 ± 0.91 *^,c^ (25.62)	11.42 ± 0.08
**AT8**	0	41.64 ± 1.12 *^,f^ (7.82)	3.13 ± 0.10
	30	39.75 ± 1.4 *^,e^ (12.02)	5.19 ± 0.02
	60	37.92 ± 1.2 *^,d^ (16.07)	7.06 ± 0.10
**SN7**	0	43.40 ± 1.8 *^,g^ (3.94)	1.68 ± 0.20
	30	39.96 ± 1.1 *^,e^ (11.55)	4.425 ± 0.01
	60	29.85 ± 0.47 *^,b^ (33.93)	15.13 ± 0.31
**MS1**	0	43.73 ± 0.4 *^,g^ (3.20)	0.64 ± 0.01
	30	43.26 ± 0.7 *^,g^ (4.25)	0.74 ± 0.01
	60	41.88 ± 1.1 *^,f^ (4.33)	1.54 ± 0.01
**ML5**	0	43.00 ± 0.9 *^,g^ (4.82)	1.79 ± 0.04
	30	42.45 ± 0.84 *^,f^ (6.03)	1.73 ± 0.01
	60	40.85 ± 0.1 *^,e^ (9.46)	2.65 ± 0.40
**G10**	0	40.68 ± 1.02 *^,e^ (9.95)	3.50 ± 0.31
	30	39.45 ± 1.1 *^,e^ (12.68)	4.67 ± 0.10
	60	37.82 ± 0.9 *^,d^ (16.28)	6.73 ± 0.10
**PT1**	0	41.01 ± 0.1 *^,f^ (9.23)	3.06 ± 0.12
	30	39.88 ± 0.41 *^,e^ (11.73)	4.39 ± 0.20
	60	33.95 ± 0.1 *^,c^ (24.85)	10.62 ± 1.02

The Mean of the OTA concentrations ± the standard deviation (% of OTA reduction) of the triplicates are represented in this table. Statistical differences are indicated as: * = significant difference (*p* < 0.01). Data with the same letters are not significantly different.

**Table 2:** OTA detoxification activities of actinobacterial strains and their corresponding supernatants.

Strains	Detoxification Activity of the Actinobacterial Strains	Detoxification Activity of the Supernatants
	OTA Concentration with Strains (ng/mL) (% of Reduction)	OTA Concentration with Supernatant (ng/mL) (% of Reduction)
Control	95.45 ± 1.7 ^a^	95.45 ± 1.7 ^a^
AT10	45.84 ± 1.05 *^,b^ (51.94)	75.51 ± 1.4 *^,b^ (20.89)
AT8	55.23 ± 0.7 *^,c^ (42.13)	74.05 ± 2.8 *^,b^ (22.42)
SN7	45.16 ± 1.13 *^,b^ (52.68)	80.10 ± 2.4 *^,c^ (16.08)
MS1	73.65 ± 0.6 *^,d^ (22.83)	79.38 ± 1.7 *^,c^ (16.83)
ML5	63.70 ± 1.13 *^,e^ (33.24)	75.69 ± 0.43 *^,b^ (20.70)
G10	59.52 ± 2.6 *^,f^ (37.65)	79.73 ± 0.7 *^,c^ (16.46)
PT1	52.52 ± 1.25 *^,c^ (44.97)	82.01 ± 1.03 *^,d^ (14.08)

The Mean of the OTA concentrations ± the standard deviation (% of OTA reduction) of the triplicates are represented in this table. Statistical differences are indicated as: * = significant difference (*p* < 0.01). Data with the same letters are not significantly different.

**Table 3:** The fungal dry weight (g), radial growth (cm) and OTA production (ng/mL) of *A. carbonarius* S 402 in coculture with AT10, AT8, SN7, MS1, ML5, G10 and PT1 on PDA medium at 28 °C for 5 days.

Strain	Fungal Dry Weight (g)	Radial Growth (cm)	OTA Production (ng/mL) (% of Reduction)
Control	3.06 ± 0.015 ^a^	4.5 ± 0.35 ^a^	14.45 ± 0.33 ^a^
AT10	3.17 ± 0.02 *^,d^	4.9 ± 0.1 *^,d^	13.73 ± 0.56 ^a^ (4.90%)
AT8	3.08 ± 0.01 ^a^	3.8 ± 0.05 *^,b^	13.68 ± 0.16 ^a^ (5.32%)
SN7	3.01 ± 0.01 ^a^	3.7 ± 0.1 *^,b^	13.90 ± 0.09 ^a^ (3.78%)
MS1	2.84 ± 0.015 *^,c^	4.3 ± 0.05 ^a^	10.99 ± 0.28 *^,c^ (23.94%)
ML5	2.75 ± 0.01 *^,c^	4.0 ± 0.02 *^,c^	12.47 ± 0.22 *^,b^ (13.70%)
G10	3.03 ± 0.01 ^a^	4.25 ± 0.04 ^a^	12.51 ± 0.09 *^,b^ (13.50%)
PT1	2.08 ± 0.01 *^,b^	2.15 ± 0.1 *^,e^	12.76 ± 0.20 *^,b^ (11.69%)

The means of the fungal dry weight (g), the radial growth (cm) and the OTA concentrations (ng/mL) ± the standard deviation of the triplicates are represented in this table. Statistical differences are indicated as: * = significant difference (*p* < 0.01). Data with the same letters are not significantly different.

**Table 4:** Normalized expressions of the genes involved in OTA production in *A. carbonarius* S402 after five days of coculture at 28 °C with AT10, AT8, SN7, MS1, ML5, G10 and PT1 on PDA.

Strains	AT10	AT8	SN7	MS1	ML5	G10	PT1
Gene	Normalized Relative Expression						
*acpks*	1.00	1.02	0.99	0.629 *	0.61 *	0.91 *	0.99
*acOTApks*	0.998	1.02	1.01	0.761 *	0.77 *	0.817 *	1.01
*acOTAnrps*	1.01	0.993	0.992	0.79 *	0.881 *	0.881 *	0.992
*laeA*	1.01	1.01	1.03 *	1.09 *	1.01	1.01	1.03 *
*veA*	0.998	0.98 *	0.9 *	0.886 *	1.00	0.93 *	0.9 *

Statistical analysis was made using the STATGRAPHICS Centurion XVI (version 16.1.11 for windows, 2010, StatPoint Technologies Inc., Warrenton, VA, USA). Data with (*) = significant difference (*p* < 0.01).

**Table 5:** List of primers used in this study.

Primer Name	Primer Sequence (5′-3′)	References	Efficiency
*acpks-F*	GAGTCTGACCATCGACACGG	[33]	95.0%
*acpks-R*	GGCGACTGTGACACATCCAT		
*acOTApks-F*	CGTGTCCGATACTGTCTGTGA	[34]	98.5%
*acOTApks-R*	GCATGGAGTCCTCAAGAACC		
*acOTAnrps-F*	ATCCCCGGAATATTGGCACC	[35]	102.1%
*acOTAnrps-R*	CCTTCGATCAAGAGCTCCCC		
*laeA-F*	CACCTATACAACCTCCGAACC	[36]	100.7%
*laeA-R*	GGTTCGGCCAACCGACGACGC		
*veA-F*	TCCCGGTTCTCACAGGCGTA	[36]	99.3%
*veA-R*	GCTGTCCTTGGTCTCCTCGTA		
*β-tubulin-F*	CGCATGAACGTCTACTTCAACG	[37]	101.1%
*β-tubulin-R*	AGTTGTTACCAGCACCGGA		

## Abbreviations

*A. carbonarius*	*Aspergillus carbonarius*
qRT-PCR	Quantitative Reverse Transcription-Polymerase Chain Reaction
OTA	Ochratoxin A
LAB	Lactic Acid Bacteria
AFB	Aflatoxin
PDA	Potato Dextrose Agar
ISP2	International *Streptomyces* Project-2
PBS	Phosphate Buffer Solution
HPLC	High Performance Liquid Chromatography
rpm	Round per minute
